# Nonhuman Primates in Public Health: Between Biological Standardization, Conservation and Care

**DOI:** 10.1007/s10739-023-09721-z

**Published:** 2023-07-21

**Authors:** Tone Druglitrø

**Affiliations:** TIK Centre for Technology, Innovation and Culture, University of Oslo, PB 1108 Blindern, 0317 Oslo, Norway

**Keywords:** Nonhuman primates, Biological standardization, Care, Conservation, Vaccines, Public health, The World Health Organization

## Abstract

By the mid-1960s, nonhuman primates had become key experimental organisms for vaccine development and testing, and was seen by many scientists as important for the future success of this field as well as other biomedical undertakings. A major hindrance to expanding the use of nonhuman primates was the dependency on wild-captured animals. In addition to unreliable access and poor animal health, procurement of wild primates involved the circulation of infectious diseases and thus also public health hazards. This paper traces how the World Health Organization (WHO) became involved in the issue of primate supply, and shows how by the late 1960s concerns for vaccine development and the conservation of wildlife began to converge. How did the WHO navigate public health and animal health? What characterized the response and with what implications for humans and animals? The paper explores how technical standards of care were central to managing the conflicting concerns of animal and human health, biological standardization, and conservation. While the WHO’s main aim was to prevent public health risks, I argue that imposing new standards of care implied establishing new hierarchies of humans and animals, and cultures of care.

In 1971, the World Health Organization (WHO) organized an international symposium in collaboration with the University of Berne and the Swiss Serum and Vaccine Institute about the breeding of primates for laboratory use. The Berne symposium was one of several meetings that would be organized by the WHO in the coming decades that addressed the problems of supply, health hazards, breeding and use of nonhuman primates in the context of the continuous decline of natural habitats of different primate species and emerging critical voices for wildlife conservation. The meeting was a follow up of the *WHO Technical Report Series, no. 470* (1971) published the same year entitled: *Health Aspects of the Supply and Use of Non-Human Primates for Biomedical Purposes. Report of a WHO Scientific Group*. In this article, I explore the work of developing the Technical Report 470 (1971). The 1971 report and the work leading up to it provide a window into how the “growing burden of disease” (Packard 2016, p. 125) that the WHO had dealt with since the 1950s was increasingly forcing the agency to engage with animal health issues. In 1967, seven laboratory workers were infected by and died of a virus after interacting with African green monkeys. The virus was named the Marburg virus after first being identified in a laboratory in a small town called Marburg in Germany and was later the same year linked to outbreaks in Belgrade, Yugoslavia and Frankfurt, Germany. The problems were thus not specific to one site but were transnational. These circumstances placed additional pressure on the WHO to deal with zoonotic diseases, and particularly so the risks of infection that followed from the circulation of wild primates for biomedical science.

Another and related context of the primate issue was the growth in dependency upon wild primates in large numbers by European and US biomedical science since the 1940s, especially rhesus macaques from India (Ahuja 2013, 2016). Noteworthy studies have shown how nonhuman primates became ‘conscripts’ of imperial projects of the US (Ahuja 2016; see also Haraway 1989; Milam 2019; Suri 2022; Guerrini 2003, 2022). The biomedical venture of nonhuman primates has been labelled “extractive colonialism and neo-colonialism” (Haraway 1989, p. 115) and “racialized political economies” (Suri 2022, p. 117). Furthermore, the “escalating role of corporate power,” to cite Bolman (2022, p. 149), in laboratory animal supply in general from the 1950s and onwards also played into this context, bringing new actors into the mix. By studying the making of beagle dogs into a “global toxicological standard” (p. 148), Bolman demonstrates the central role of corporate actors in “producing (breeding) laboratory organisms as commodities (‘biocapital’) and shaping their international networks of circulation and exchange” (p. 149).^1^ These studies are important because they show how humans and nonhuman animal lives are tied together in ways that have certain political, cultural and normative implications, and that are situated in time and space.

Parallel to the body of studies pointing out the colonial and neocolonial character of the primate trade in particular, as well as the commodification of animals for science in general, historians of science and medicine have shown how new standards of care in animal research became increasingly important from the 1950s. This was a particular form of “care”, attached to specific skills and expertise, sites and infrastructures, and subsequently to the legal and regulatory domain (Kirk 2016; Druglitrø 2018).^2^ “Care” involved, for instance, proper animal facilities with barrier systems, and trained staff that would know how to contain disease and promote healthy animals. While these were standards emerging initially from within the sciences due to the demand for more reliable animal material for research and for reliable procurement of animals, “care” in animal research was also increasingly linked to public concerns for animal welfare and good care became key to the moral justification of animal suffering (Kirk 2019; Kirk and Ramsden 2021; Druglitrø 2014, 2018).

This paper explores the history of nonhuman primates in science and public health in a context where biological standardization and vaccine development were established as key to combating epidemic diseases. It focuses on how the primate issue unfolded and was worked upon in the specific context of international public health and the WHO in the late 1960s and early 1970s. The aim is to provide a better understanding of not only how problems of animal health became tied to issues of public health in this period, but also of how challenges that are deeply political and cultural became encapsulated in technical responses to health. The questions guiding the analysis are: How did the WHO navigate public health and animal health? What characterized the response, and with what implications for humans and animals? To answer these questions, the paper engages with what Martin et al. (2015) have called “care’s darker side: its lack of innocence and the violence committed in its name” (p. 627). Placing focus on the darker side of care practices and strategies, Martin et al. (2015) also bring attention to how power relations are enacted in “on-the-ground sites of care” (p. 626). The “care” that was formulated in and promoted by the Technical Report 470 was more forceful and “slippery” (Martin et al., 2015, p. 626) in normative terms. As this paper will show, managing the issue of primate supply in the context of the WHO—more specifically the Unit of Biological Standardization and the Unit of Veterinary Public Health—shaped the issue in a certain (technical and scientific) way. While care practices, infrastructures, and expertise were seen as means to reduce the hazards of using wild primates and to conserve natural habitats that were facing depletion, it also produced new standards for using nonhuman primates that stripped them of culture and enabled a critique of specific versions of care cultures and human-animal relations. The report, thus, serves as an important and very concrete site in which nonhuman primate bodies and human-non-human primates' relations were (re-)enacted and innovated upon in the context of health risks and public health. As Asdal (2015) has shown in her work on cod and aquaculture, formal documents can be analysed as devices that work upon bodies for economic, political, and/or scientific purposes. Documents do not only reflect reality, but also (potentially) act upon it and modify its organisation.^3^ As I will show in the following sections, stakes were attached to the report in its entirety, as well as the specific formulations and ways of framing the issue of primate supply and use in biomedicine. Given the dependency on nonhuman primates in biomedical science, and in vaccine development against emerging viruses in particular, much faith was placed in the WHO proceedings and report to developing internationally shared standards.^4^

The ensuing analysis is organized as follows: I start by teasing out how the issue of primate supply was taken up and handled by the WHO and how the agency framed and approached the problem. In this first part, I focus on how the Unit of Biological Standardization (BS) and the Unit of Veterinary Public Health (VPH) tried to navigate the issue of wildlife conservation in the context of public health. These discussions reflected and enacted who and what is relevant for and is to be given care within the context of public health at the time. Following from this, I attend to the procedures of preparing the Technical Report and the specific scientific and technical expertise that was drawn upon to develop a draft and, consequently, show how the scientific community responded to the draft prepared by the WHO expert group. Here I tease out how tensions and disagreement existed in the scientific community on how to approach the convergence of vaccine development and conservation issues. I also demonstrate how the primate field—while embracing a broad approach to the problems of primate supply, including concern for animal health and economic differences—established essentializing distinctions between importing and exporting countries based upon standards of care and expertise established in European and American laboratories.^5^ In the last parts of the paper, I discuss the outcomes of the discussions and negotiations in the WHO as they were formulated in the final Technical Report, with a special focus on how breeding in captivity was articulated and contextualized, and how it established new hierarchies of good and bad laboratory primates, and good and bad cultures of care.

## Nonhuman Primates, Conservation and Public Health

The question of “supply, safe-handling and use of nonhuman primates in biomedicine” (WHO 1969, p. 26) was first formally discussed at a week-long meeting by the 21st Expert Committee in Biological Standardization at the WHO headquarters in Geneva in fall 1968. In January 1968, the WHO had received a formal request from the Committee of Scientists for the Use of Primates in Medical Research in the US. The request was that the WHO should convene a meeting for a scientific group “as soon as possible to examine the problem in detail and recommend measures designed to ensure the continued supply of [nonhuman primates] for the welfare of man and also for the conservation of the species” (Goldsmith 1968, p. 1077). This resolution by the Committee of Scientists for the Use of Primates in Medical Research in the US to approach the World Health Assembly—which is the decision-making body of the WHO with health ministers from member states—was published in *Science* the same year, with the stated aim of raising the issue of primate supplies on an international scale due to the continued threats of embargo from exporting countries. Following up on the request from the US scientific committee, the 21st Expert Committee in Biological Standardization convened a group of experts in primate medicine to develop a set of technical recommendations for responding to the hazards linked to the primate trade and supply.^6^ The report from the meeting read,

In view of experiences in the spread of infection from non-human primates, the Committee agreed that there was a need for recommendations for the guidance of laboratories in which non-human primates and primate tissues were used. The Committee also agreed that it would assist in the formulation of such recommendations, especially in relation to the manufacture and control of biological products. The Committee emphasized however, that it was desirable that the recommendations should be drawn up so as to cover adequately the many spheres of interest concerning non-human primates, and that arrangements should be made for distributing such recommendations widely. (WHO 1969, p. 26)

The main work, as the Unit of Biological Standardization (hereafter BS Unit) saw it, was to translate the challenges related to the primate supply into a technical concern that could be solved mainly by a technical response. For instance, the issue of embargos on the export of primates would not be addressed; the focus was rather placed on the scientific and technical measures to respond to zoonotic diseases and other health hazards related to the trade, aspects that were also particularly relevant to biological standardization. As a consultant for the BS unit would later write: “The BS programme has a direct interest, related to the safety of a number of biological products, in ensuring that healthy nonhuman primates are available for the preparation and testing of such products.”^7^ The Chief of Veterinary Public Health Unit (hereafter VPH Unit), William Beveridge, wrote to his colleague Walton Ted Roth about the initiative to develop technical recommendations within the frames of the Biological Standardization Unit: “Recommendations by this Committee do carry a lot of weight.”^8^ Beveridge had for years been engaged in the problems of primate supply and had spent much time considering the topic of immunosuppression in order to control infections in primates. In 1969, he and H. Balner published proceedings from a conference in the Netherlands in 1968, organized in part by the VPH Unit at the WHO, dealing with this very topic and pointing to an uncontrollable supply chain as a key reason for the recurring epizootic and zoonotic outbreaks following from interactions with nonhuman primates. During the summer of 1969, in his position as the Chief of VPH Unit, Beveridge wrote two memoranda (both dated 29th July 1969)—entitled *Conserving Future Supply of Primates* and *Primate Recommendations*—which he sent to the BS Unit for consideration. The background for the first memorandum was an ongoing effort to establish collaborations with the Food and Agriculture Organization (FAO). H. H. Roth, a Wildlife officer at FAO wrote to Beveridge in the summer of 1968:

As I have mentioned to you, we are in touch with various wildlife groups which all have an interest in primates, one way or the other. Since the interest of WHO in this subject is restricted to the public health aspects it might be useful, if our two organizations maintain close working relations in the wildlife field in general, particularly with a view to the exchange of information.^9^

The Chief of the BS Unit, Aubrey Outschoorn, replied to Beveridge’s memoranda, “I am personally uncertain that the question of supply of primates, in the context of conservation of animal life, is a matter for WHO.”^10^ He also revealed to Beveridge that there had been disagreement at the meeting of the WHO Expert Committee on Biological Standardization meeting in 1968 on how to situate and make the problems of primate supply relevant to public health as defined by the agency. Conservation and supply were only of “secondary relation” to biomedicine, Outschoorn stated, and continued,

The 21st Expert Committee was extremely loath to include a firm recommendation [to address the issue of primate supply] in its report (Wld Hlth Org. Techn. Rep. Ser., 413, 26) and only did so under considerable pressure.^11^

Outschoorn wrote to Beveridge several times during 1969 regarding this issue, making his contention of considering this matter within the frames of the WHO expert committees:

I do not see whether it would be proper for WHO to be concerned with the preservation of wildlife as there are other bodies which may be interested in this aspect. The only grounds on which I feel WHO could enter the area would be from a public health point of view, namely, if non-human primates became less available, vaccine production and control could be interfered with.^12^

The draft of the Technical Report was in the end written in collaboration between the Unit of Veterinary Public Health and the Unit for Biological Standardization. The introduction to the report provides an interesting view of how the expert group chose to describe and frame the current situation and the role of nonhuman primates. The section described how monkeys had increasingly become important biomedical objects in the last 15 to 30 years. The reason for this increase was the development of vaccines against the poliomyelitis virus, which was a known success story. Unfortunately, the section read, a side effect had emerged with this expansive and at times uncontrollable traffic in nonhuman primates: there was a wastefulness in the supply chain of wild primates. Animals were discarded before reaching the plane to the lab, many fell sick, and very often they would die before or on arrival in the quarantine station in the importing country. The problem was said to be located mainly in the exporting countries and with shady actors in the primate trade. The Report was organized according to chapters focusing on implementation of the recommendations, special problems (which were feeding, transmission of disease, tuberculin testing of primates and injuries), conservation of supplies of monkeys and apes, breeding in captivity and the training of personnel. These chapters addressed general concerns and advice related to the supply and use of primates. The technical, and more specified, recommendations were placed in the annex of the report and were organised into four chapters (with subpoints): Holding and Exportation; Transportation by Air; Health Aspects Related to Biomedical Use; and National Control. The chapters were formulated as action points to affect the development of “appropriate measures” and to “contribute to the safer and more effective use of monkeys and apes for biomedical purposes” (WHO 1971, p. 6). What were the appropriate measures according to the experts at the agency? How did the draft report navigate the competing concerns?

## The Infectious Wild Primate, and Good and Bad Human-Primate Relations

The draft recommendations relied heavily upon a report ordered by the BS Unit from Frederic T. Perkins in the preparatory phase. Perkins was the Head of the Division of Immunological Products Control, National Institute for Medical Research in the UK, and received an invitation to write an expert report to the BS Unit about the primate procurement problem. He would later be part of the scientific committee writing up the final version of the Technical Report 470. In his scientific report for the draft recommendations, entitled “The Housing and Handling of Monkeys to Decrease Transmission of Disease to Man,” Perkins focused on the nature of the experimental animal and relations between infections and health standards. He started his report by writing:

All monkeys must be regarded as highly infectious and must, therefore, be housed and handled in such a manner that transmission of disease to man is reduced to a minimum.^13^

Perkins continued to identify tuberculosis, salmonellosis, shigellosis, herpes virus simia (B virus) and the Marburg virus, which had so far looked like a haemorrhagic virus, as key agents in causing infectious diseases by the handling of wild primates. In addition to these viruses, other simian specific viruses were of potential, yet unknown, danger to human health. Because of these known and unknown risks, the control of disease could only be achieved by having a well-equipped and staffed animal house, adequate space, good barrier system, nursing and sterilisation techniques, suitable protective clothing and good diets.

Like other laboratory animals, the issue of husbandry and care was formulated in a scientific and technical manner: animal health was defined according to the absence of disease and reduction of stress. Perkins referenced the standards for laboratory animal care and husbandry developed by the Institute of Laboratory Animal Research (ILAR), named Laboratory Animal Quality Standards. For primates, he advised an additional rigid testing regime to ensure effective identification of infectious diseases during the quarantine. To do this, monkeys should always be sedated in order to “reduce response to environment, reduce aggressiveness, and produce recumbent immobilization.”^14^ This advice was closely linked to how wild-captured monkeys were viewed, as demonstrated by Perkins: as biohazards endemically housing dangerous pathogenic diseases, and that needed to be handled as such. He devised a plan for sampling and tuberculosis testing and proposed different testing regimes for “transient colonies” and “maintenance colonies”—that is, those colonies where monkeys were only housed temporarily, and those that were housed to be maintained, used, and bred upon on site. In this detailed account, he described the necessary intervals in which tuberculin testing should be done, and when monkeys could be “certified as tuberculosis-free.”^15^ Summarizing the main purpose of the recommendations, he wrote:

The purpose of these recommendations is to improve the conditions under which nonhuman primates are captured, transported and conditioned in laboratories in order that more healthy animals will be available for vaccine production, safety tests and research.^16^

While Perkins’ report was concerned with conditioning nonhuman primates into the laboratory context and separating the animals from culture, so to speak, the report ordered by the VPH Unit to investigate the situation in the exporting countries was concerned with demonstrating how culture interfered with nature (and science). The VPH Unit ordered a report from a scientist with experience in the field, Walton T. Roth.^17^ Roth emphasized a qualitative and practical difference between standards expected from scientists in (Western) laboratories and those of the trappers in the exporting countries. He wrote: “Methods used today to obtain primates for sophisticated biomedical research are essentially the same as those used during the stone-age to trap food.”^18^ Using his experiences from India as an example, Roth wrote:

The most frequently used species, M. mulatta, is trapped predominantly in the drainage area of the Ganges River which is severely polluted by human waste products. C. aethiops is ecologically classified as a ‘gallery forest dweller’, i.e. lives mostly along creek and river basins replete with waste products of all the human settlements upriver from their habitat. Saimiri, Papie and others must drink water to survive; so must people who usually build their villages on or near a stream.^19^

He continued to contrast this state of affairs to what he viewed as “sophisticated biomedical research,” by emphasising the complex role of nonhuman primates in the countries in which they were captured—being at the same time pests, cohabitants and sacral beings. Interestingly, Roth expressed little concern for the health of the humans that lived in conjunction with nonhuman primates at the edges of cities, forests and river areas. On the contrary, he contended that the conceptions of sanitation, hygiene and husbandry were the very cause for laboratories receiving fragile primate bodies. Roth explained that the “biological physiological relationship to man render primates as excellent receptors for most all human pathogens as well as many other zoonotic agents encountered in the wild.”^20^ Due to the modes of living in the exporting countries (read: India), the situation for nonhuman primates were “beyond control in many areas of primate origin.”^21^ Taken together, the Perkins report and the Roth report amplified the view that the problem of hazardous primates was located with the exporting countries and unsuitable practices and infrastructures there. Roth’s account supported Perkins’ technical framework for ensuring healthy primates and for preventing the spread of diseases—even if only indirectly—by reinforcing the view that the problem was embedded in improper human-primate relations and standards of trapping, housing and care in the exporting countries.

In the following sections, I set out to describe and analyse the comments and responses from the broader scientific and corporate community to the draft report that was circulated to the international scientific community, industry and corporate actors, and public health authorities in early 1969. The responses were both critical and affirmative of the ways in which the report was framed. Investigating the responses in detail provides a deeper understanding of how different concerns were being linked together and problematised by different actors, and the means and strategies suggested as necessary for resolving the problems related to primate trade and procurement. As we will move to in the next section, the discussions revealed a scientific community that disagreed on how to approach and think about the relationship between animal health and human health, as well as a scientific practice—primate-based research—that was facing increased public criticism (Haraway 1989) based upon concerns for animal suffering and the loss of wildlife. These concerns were closely tied to the wordings and formulations in the report, but also to the overall philosophy it presented.^22^

## Practices in Conflict: Vaccine Development and Wildlife Conservation

Letters with comments on the draft report and the recommendations began to arrive at the WHO offices in Geneva in March 1969, shortly after the report was circulated. The BS unit hired a consultant, D.G. Evans, to write a report synthesising the comments and singling out the main points. Evans reported on the reception of “numerous replies covering many different fields of interest.”^23^ Comments from US-based scientific actors and institutions dominated the collection of replies, as well as reports and comments from big corporate actors, such as the Pfizer group and Shamrock Farms. The relationship of scientists to the critical public and the public reputation of experimental medicine, as well as the reputation of those involved in the monkey trade were expressed as major concerns by many of the US-based actors.^24^ The concerns were linked to the introductory text to the report, where they felt that the scale of death was exaggerated or emphasized too much. Bernard F. Trum, Director of the Animal Research Center at the Harvard Medical School, wrote:

For example, under General Considerations, mention is made of the large ‘proportion’ of animals which die. I presume this means a large number! But without data, I think it is a bad statement and suggest again that if it could be said better and protect the reputation of those who are assisting us in procuring animals.^25^

According to Trum, formulations and wordings were key, as “we have noticed that adversaries often pick up certain of our statements to be used against us.”^26^ Arthur Riopelle^27^ from the Delta Regional Primate Research Center in the US also criticised the focus on the “magnitude” of the primate trade and the focus on the scale of death of monkeys due to trade and infectious diseases, writing:

I would question the magnitude. I would also question the appropriateness to express this concern without true facts. I am questioning the statement: ‘A large proportion of animals die or are discarded between trapping and receipt, etc.’ While it is true that numbers of these animals are selected out, or perhaps die, the magnitude is not sufficiently known to express it as ‘large.’^28^

Other focuses were on who and what the report was meant to protect or target—that is, what were the main objects (or subjects) that the report was aiming to care for? For instance, Riopelle questioned how the recommendations in the report ultimately served or represented *public health*:

[T]he purpose of the recommendations is stated as a) to improve the health status of monkeys, etc. and b) to minimize the risk of infection to personnel. It seems to me that they have the a) and b) reversed. It is my impression that this document relates to the public health aspects of handling primates and the justification for making the changes in recommendations which are stated and is based on the threat of disease transmission to the humans.^29^

Riopelle asserted that the report should reflect “a stronger attitude toward the problems and efforts to eliminate public health threats.”^30^ He claimed that in formulating the recommendations related to public health, it was irrelevant to place too much focus on wildlife conservation and animal supply. In his view, depletion should only be considered relevant if it affected biomedicine’s access to animals or if dangerous pathogens could transmit from primates and hence represent professional and public health hazards.

The strict focus on public health, and how health risks should mainly be emphasised in relation to the humans working with these animals, was however challenged by other scientists, such as the director of Rijksinstitut voor de Volksgezondheid in Utrecht, Netherlands, H. Cohen, who disagreed with Riopelle’s positions on this topic. He wrote, “I would like to comment on the underlying philosophy of the document and the form wherein it is written.”^31^ He continued to write:

[I]t is in my opinion that these recommendations should take into account the growing threat to many species of primates due to the enormous drain on the supply by the present need in laboratories all over the world […] This problem should be stressed in the document not only for the purpose of protecting wildlife but also to ensure continuation of supply. This might in the future lead to reconsideration of existing requirements especially those of virus vaccines with the aim of replacing primary monkey tissues by other primary cell strains and to avoid the use of monkeys for control purposes where this is possible.^32^

Similarly, the Wildlife Officer of the Animal Production and Health Division of the Food and Agricultural Organisation of the United Nations (FAO), H. H. Roth, commented that the conservation of monkeys was just as relevant to public health as was the use of monkeys in laboratory work. He pushed for a parallel concern for the conservation of monkey habitats as Cohen did, while also establishing the primate issue as a mutual concern for the two international organisations:

The general subject of primate traffic is of considerable interest to FAO as well as WHO. Whereas FAO is interested mainly from a wildlife management and conservation point of view, WHO is only interested in the serum production and public health aspects. However, in my opinion it is essential to see the different aspects in conjunction and to get the respective professional groups to cooperate. In other words, public health legislation, quarantine regulations, etc., would help to regulate the off take of monkeys and enable conservationists to step in if necessary. On the other hand, conservation legislation is, of course, of great importance to public health too, because it ensures sustained availability of laboratory monkeys and supports import control.^33^

The position presented by FAO, here represented by wildlife officer Roth, thus strengthened the link between conservation and reliable procurement of primates, and in the same move established animal health issues and public health issues as integral to one another. The primate issue was in other words established as a concern that permeated the boundaries of one international organisation or one set of expertise, and forced the WHO to expand the scope of their engagement in animal health.^34^ The primate issue would become a key collaborative concern for the WHO and FAO following the report, where veterinary public health would play a central role in their ability to manage and work upon health threats (and food security issues) in the interface between humans, animals and the environment.

The final report refined the language describing both the scale of primate deaths related to biomedical science (changing the wording from “a large proportion of animals” to what was considered as a less dramatic formulation: “many animals” die between trapping and arrival at the designated animal facility) (WHO 1971, p. 16) as well as the relationship between conservation, use, and a more predictable and safe supply of nonhuman primates. The topic of conservation would be addressed, but the draft did not contain any technical recommendations that targeted conservation practices. It is however interesting to note how conservation would emerge continuously in the report as a positive side effect (WHO 1971) of the technical recommendations targeting procurement, housing, and care. In the following, I explore in more detail how the technical recommendations in the draft report were responded to by stakeholders and how distinctions between importing and exporting countries were established based upon the quality, ability and availability of expertise and care of nonhuman primates.

## Translating Standards of Care Between Importing and Exporting Countries

While the prospects of the report offending or further agitating the critical public was a key concern for many US-based stakeholders, it was the technical standards related to infrastructures and expertise that emerged as the most contested point of the circulated draft. The recommendations focused on the need to ensure better national regulation and control of the primate trade to prevent the spread of infectious diseases, and to do so by establishing new standards for the holding, care and exportation of primates. Indeed, the comments from the stakeholders reflected a demand for shared standards and regulatory measures. However, they diverged in terms of what measures were deemed the most effective and, not the least, translatable across importing and exporting countries. Perkins’ report dictated the technical standards recommended in the draft report. These recommendations targeted, as listed above, standards of care and expertise oriented around disease risks and hygienic measures related to the housing of primates in laboratory settings. Perkins relied heavily on established standards of husbandry and care of organisms that represented biohazards, like ILAR, as well as his own experience from working with primates at the National Institute for Medical Research Institute in the UK.^35^ Although representing the latest standards, the requirements for infrastructures and expertise as suggested by the recommendations were regarded as unattainable and impractical by many of the respondents. As care for laboratory animals was closely tied to specific technoscientific arrangements, skills, and expertise, it was also a radically situated form of care, emerging from a specific culture, so to speak. H. H. Roth, again representing FAO, commented that the recommendations “sound somewhat too ambitious, almost academic.”^36^ He questioned if the recommendations would have any impact at all due to the formulations, and because local authorities and individuals in exporting countries would find them “hopelessly unrealistic.”^37^ Comments were also made about the qualitative difference between the exporting countries and the importing countries in much the same vein as T.W. Roth had done in his report to the WHO. The British oral surgeon, W. H. Bowen, for instance remarked:

While I agree with many of your recommendations relating to the exporting country, I would not be very optimistic about having them implemented, particularly when one considers that you are asking that the animals in all probability be housed in conditions better than those which the handlers’ experience.^38^

In a similar manner, L. H. Schmidt, professor in pharmacology at the University of California, Davis, wrote:

In theory the ‘holding and exportation’ practices set down in this section are good. In practice they may be unrealistic and non-attainable. Most nonhuman primates are found in the developing or less well-developed countries where physical facilities or the wherewithal to develop them, and technically trained personnel are in short supply.^39^

Correspondingly, Geoffrey Bourne, Director of the Yerkes Regional Primate Centre, described what he regarded as the absence of proper infrastructures and expertise for housing and quarantining nonhuman primates in the exporting countries:

In my experience in Thailand the facilities for holding animals are not good; they were better in Malaya. One of the best dealers I investigated receives animals brought in by trappers in a large building which simply consists of a palm thatched roof supported by posts and with a concrete floor. There are no walls [...].^40^

Others made an effort to place the trappers and dealers in a more favorable light, pointing to the economic dependency on *live* monkeys, like representatives of the Hebrew University Medical School in Jerusalem:

It is most difficult to expect countries short of medical personnel (even for the needs of the local population) and without any veterinary services to be able to provide proper guidance and assistance in caring for animals that are to be quarantined, treated and looked after for some time before being shipped abroad[…] Nonetheless, one got the impression that dealers did whatever they could, within the limits of their knowledge, to take good care of the monkeys, and for the obvious reason that only live monkeys could be shipped out.^41^

Most comments on the draft were united around the suggestion to relieve the exporters of primates from tasks such as quarantine and testing that would require specialized infrastructures and scientific expertise. Corporate actors were particularly welcoming to the involvement of the WHO in the problems of primate supply. H. Balner from the Radiobiological Institute in the Netherlands was literally enthusiastic:

I have read it with great interest and enthusiasm. Virtually the entire draft reflects in general lines the thoughts we, who buy and use primates on a large scale, have often expressed. It is therefore gratifying to see that you are actually going to do something about it. Hopefully on a world-wide basis.^42^

Corporate actors also pushed ideas about the establishment of quarantine facilities and collection depots in the ‘exporting countries’ that could be headed by what was regarded as “qualified experts.”^43^ Several suggested a system where monkeys were “trapped to order.”^44^ The Pfizer Group wrote, “if a constant level of supply is needed on a year-round basis it is not always possible to catch monkeys at the optimal season.”^45^As Shamrock Farms suggested, “trapping presents some difficulty in so far as stipulating any particular time limit and this could really only be done satisfactorily if all animals were trapped to order.”^46^ To realise this ideal, the establishment of “centralised depositories” or a “central collecting depot” of monkeys would ensure that the supplier at most times would be able to meet the specific needs of the user.^47^ The depositories were also said to affect (or contain) the temporalities of natural habitats, as they would ensure “sustainable drainage of resources.”^48^ The depositories would serve to overcome seasonal variations that affected the health status of the primates. A key problem, however, was getting national governments onboard. As H. Balner continued to write in his letter, putting his enthusiasm aside:

[H]ow is it all going to be implemented and financed? Even if you have the cooperation of the more advanced exporting countries, such as India and Pakistan for instance, do you think that it will work? Will you get the point across that national “industries” are really at stake? Some of the measures you propose require enormous investments; the quarantine stations will have to be actual veterinary laboratories, staffed with people of the caliber that we employ in our highly specialized laboratories.^49^

A. K. Thomas, the Director of the Central Research Institute in Kasauli, India, confirmed the instigation by Walton T. Roth about the primate trade being out of control, commenting in April 1969 that until recently there had been little “official supervision” over transport and holding of nonhuman primates—particularly the rhesus macaque, of which India was a huge exporter. Export was primarily organized by private companies where the “standards of care” were insufficient, Thomas wrote, and pointed out that inspections on practices and infrastructures of holding and care by representatives of the importing countries would be imperative to raising the quality of the monkeys.^50^

The final Technical Report highlighted “adequate national control measures” (WHO 1971, p. 25) in exporting countries as key to managing unreliable access and supply, as well as health hazards of handling wild primates. “Until recently” the Technical Report read, “neither the public health nor the veterinary authorities in most countries has been much concerned with the health aspects of international trade in, or use of, primates” (p. 5). There was thus a desperate need for “appropriate facilities and official machinery for controlling the export and import of monkeys and apes” (p. 19). This involved organising a “healthy market with serious and certified market actors” to control the supply of primates, and to ensure that quarantine, care, and husbandry practices met biomedical standards. Nevertheless, the report read that “any improvement, however small, would contribute towards reducing the risk of transmitting disease to man” (p. 19), and in this way acknowledged how availability to nonhuman primates for biomedicine was caught in a complex web of social, technical, economic and political differences, as well as questions of population of species and nature conditions. How could these differences be overcome? How did the report navigate the different concerns and situations of importers and exporters?

## Breeding International Standards: Good and Bad Laboratory Primates

While breeding of nonhuman primates was not the subject of the Report, it was given its own chapter (Chapter 5). In addition, the conclusion of the report pointed to future activities which targeted the prevention of losses, ensuring future supply and public health measures—thus drawing together conservation concerns with public health concerns as defined by the WHO. Internally in the WHO, the BS Unit organized the mapping of how nonhuman primate supply and use was relevant across the different technical units of the agency. The result from the mapping emphasized the broad application of nonhuman primates to biomedical fields, ranging from: comparative studies of human diseases, the production and testing of biological products, research on viral diseases, drug dependence testing, the testing of drugs for teratogenicity, reproductive physiology, toxicity and food additives testing, general and applied immunological research, routine purposes such as harvesting organs for tissue culture, occupational hazards for personnel handling primates and their tissues, cardiovascular diseases, cancer, behaviour and mental health, malarial parasites and treponematoses.^51^ Furthermore, many of the respondents to the draft recommendations had emphasized the need to differentiate better between different primate species. Dr. D. Ploog at the Max Planck Institute commented for instance how the recommendations for quarantine and care were very much targeting different macaque species.^52^

In his report, and drawing upon the comments they had received, Evans emphasized the breeding strategy as “the most satisfactory and ideal method for ensuring that healthy nonhuman primates are available for biomedical purposes,” and “to breed from healthy females.” He wrote that while there are primate centres in “considerable number” throughout the world, these centres were not able to meet all demands for biomedical work and particularly so in the context of “manufacture and testing of viral vaccines and other pharmaceutical products.”^53^

In late September 1970, a group of scientists from US, UK, Uganda, India, and the Soviet Union as well as representatives from ICLAS (Erichsen), International Office of Epizootics (Vittoz) and International Union for the Conservation of Nature and Natural Resources (Holloway) had prepared and finalised the Technical Report 470 at the WHO headquarters in Geneva. Hence, those that were invited to be part of the Scientific Expert Group to finalise the report represented the major users and the major exporters of what was regarded as key primate species for the biomedical sciences in the past and for the coming future (such as green monkeys [*Chlorocebus sabaeus]*, rhesus macaque [*Maccaca mulatta*] and cynomolgus macaque [*Macacca fascicularus*]). While the IUCN was represented at the meeting, the final report would state that conservation would only be of secondary concern after the safety of personnel working with primates and the quality and health of animals. Conservation was said to be cared for by a firm emphasis on the need to reduce “wastage of animals from disease” (WHO 1971, p. 6) and embark on breeding on a larger scale (as had been decided in the 22nd Expert Committee on Biological Standardization meeting in 1969 after receiving the comments from different stakeholders). The involvement of conservationist actors such as the IUCN was key because the biomedical community depended on broad collaborations to manage the problems with capturing and transporting wild primates. Notably, the IUCN was operating parallel to the WHO proceedings developing the Draft Convention on the Export, Emport, and Transit of Certain Species of Wild Animals and Plants where the topic of the depletion of primate habitats was included. A key aim for the WHO was to be able to sustain the import of wild primates until breeding in captivity was properly in place (which was acknowledged to be a slow transition), and thus conservation was key to preventing future sanctions, such as embargos on wild primate trade, from the exporting countries. In the final recommendations of the Technical Report 470, *breeding* represented the only viable and best choice because it provided better animals. It stated:

The Group agreed that breeding programmes should be started without delay. An additional and important reason for the establishment of breeding colonies is the superior quality of animals bred in captivity. It was found some years ago that laboratory-bred dogs and cats were far superior to stray animals for experimental purposes. (WHO 1971, p. 11)

Hence, the Report established captive bred primates as organisms with “superior quality,” aligning primates with other experimental animals and the work it took to transform them into “truly reliable tools,”^54^ Several of the comments had focused exactly on what constituted a good experimental primate organism. L. H. Schmidt wrote in his commentary that the source of supply of primates is the “basic obstacle to procurement of healthy animals presenting fewer hazards to the end user.” He continued writing:

As I see the history of laboratory animal use, rodents and dogs became truly reliable tools for many experimental uses only with the development of techniques for rearing these subjects under rigidly controlled conditions. Few investigators would think of pursuing studies with wild caught mice, rats or rabbits; many reject street dogs as experimental tools; some but fewer take the same position with respect to cats; wherever possible aviary reared birds are employed. The same attitudes should guide the use of sub-human primates if in truth we wish to preserve the species, reduce the hazards to animal and man, and gain the most from the time and equipment of the investigator.^55^

The constant development of new scientific techniques was said in the Technical Report to make breeding of primates in captivity more feasible in the coming years and combined with substitution of primates with other animals or with tissue cell lines, conservation could also be achieved. Controlling primates with breeding techniques, mapping work and collection of information was thus regarded as central to sustaining experimental medicine and public health. The Report even made calculations of the financial costs of breeding versus using wild-caught animals, concluding,

[…] the use of laboratory-bred animals may even effect a saving in cost, in view of the enormous wastage due to contaminant viruses, which are inherent in monkey-kidney tissue and may be present in as much as 50 % of tissues from captured animals. (WHO 1971, p. 12).

While a captive bred monkey would cost more to produce compared to a wild caught one, the total gains of using laboratory animals exceeded the drawbacks. The cost of wild-caught animals was estimated in 1975 at one-tenth of the cost of animals bred in captivity (ICLAS 1976). Furthermore, a positive side effect, according to the WHO, was how these goals served conservation goals as well, and in a very particular way: breeding gave hope for conserving human-monkey futures *and* signalled care for the future use of monkeys by preserving them as natural resources.

## Conclusion

What were the outcomes of the WHO Technical Report 470 and the work to develop the report? Internally in the WHO, the Technical Report provided a foundation to further the work to organize and standardize the accessibility to healthy nonhuman primates. In 1975 and 1976 the threatening shortage of suitable monkeys for biomedicine was addressed by the World Health Assembly who drew up a scenario where this shortage “could lead to a lowering of safety standards for drugs and vaccines, as well as handicapping medical research in several disciplines” (WHO 1975, p. 1). The World Health Assembly approved a resolution “urging Member States to promote the rational conservation and utilization of nonhuman primates… and to encourage simian breeding programmes” (ICLA 1976; pp. 24–25, WHO 1976). Hence, with the technical report, we begin to see the convergence of concerns for vaccine development and the conservation of wildlife by way of breeding standardised animals and the fostering of a certain culture of care. The conclusion of the Technical Report was that the best solution was to breed primates in captivity. The decisions to target the breeding of primates in captivity as a means to transition away from the dependency on wild primates and to have access to reliable and hazard-free primate organisms can be seen as a shift in the context of primate-based research that was closely linked to the shift already taking place with smaller experimental organisms in terms of standards for quality and care. Furthermore, it also marked a shift in terms of thinking about the integral relationship between animal circulation and use on the one hand and caring for wildlife conservation as part of public health on the other, with particular attention to the convergence of biological standardization and conservation. As hinted in the introduction, the report can be described as a device (among other devices) that innovated upon nonhuman primates (see Asdal 2015) by consolidating human and animal health by way of care and breeding practices as well as encouraging intense knowledge production and collaboration across science, governments, and market actors to enable a stable access to these animals.

Furthermore, tracing how the problems related to the supply and circulation of nonhuman primates were responded to by the WHO experts has allowed me to tease out how an international public health organisation that was built to work on an international scale responded to the problem of animal health and conservation. This has revealed an agency that was both deeply biased in its approach to the problems of primate supply and limited by the logic of technical responses to endemically racial, cultural, political, and economic frictions. I have teased out how care and captive-breeding emerged as key to the technical response of the WHO as a way of caring for public health, the public reputation of the biomedical sciences and for animal health. Care in technical terms, as well as breeding, were thus tools to eliminate that which was seen as the propellers of health hazards in terms of infectious diseases. Hence, infrastructures and expertise of care were increasingly established as key to removing culture from the animals, yet themselves embodying and representing an emerging culture of care in animal research that was very much based in values and standards emerging in European and American laboratories and scientific communities.^56^ Thus, the emerging standards of care, quarantine, and captive breeding developed for the procurement and use of nonhuman primates, as explicated in the WHO report, represented a version of care that also effectively developed new hierarchies of good and bad human-animal relations. By showing these developments and by making these claims, this paper has added new empirical insight and analytical perspectives to existing studies on the supply, circulation and use of nonhuman animals in biomedicine in this period in time—a period when the very discipline of laboratory animal science was emerging as an important support science for the biomedical sciences, while becoming transnational in its scope. Last, this paper has emphasized how human-nonhuman primate relations prompt questions and investigations into what lives and human-animal relations are nurtured in international public health, and by what specific material and conceptual means others are denounced.^57^

## Footnotes

1 See also Kirk and Ramsden (2021), Kirk (2008, 2018), and Rader (2004) for noteworthy studies of the circulation of dogs and mice for science. See also the seminal anthology by Clarke and Fujimura (1992) on the development of model organisms as the “right tools” for the life sciences. 

2 In a study of the controversy around the Silver Spring monkeys in the US in the early 1980s, Kirk (2008, 2019) shows how networks of circulation of animals for science has been inscribed with and shaped by distinct and competing versions of care. His analysis demonstrates that care is attached to specific sites, practices, and technical set-ups, and thus what ‘care’ includes and who it embraces is contingent upon these variables—or material-semiotic networks. Put succinctly, he writes,“care can only be properly understood within its historical context” (2019, p. 33). See also Svendsen and Koch (2015) for a study of moral controversy around research monkeys in Denmark in more recent times, in the early 2000s, and how care was a central practice in this controversy.

3 See also Asdal and Reinertsen (2022) who have emphasized in their work on document-oriented practices that documents are sites where we can trace how issues and events unfold and how they change over time, as well as map out how norms and scales are negotiated and enacted.

4 While Haraway (1989) has shown that nonhuman primates had already been transformed into global experimental organisms’ as the biomedical primate trade was made integral to the colonial and postcolonial projects of the US, tracing the involvement of an organization working on public health issues internationally allows for an expansion of this history. Furthermore, the strong focus on zoonotic diseases and the hopes attached to vaccine development emphasized how nonhuman primates were becoming key tools for public health, also in planning for the future of biomedicine. In the same period as the WHO engaged with the primate problem, national public health institutes that had not yet had a tradition for using and keeping nonhuman primates begun to import nonhuman primates with the direct purpose of using them in vaccine research and development (see for instance Druglitrø 2016). This shows how nonhuman primates were becoming a standard organism in public health laboratories on an international scale that were not common before the 1960s. See also Nitsan Chorev's (2012) for a historical and sociological study of the transformations of the WHO in the 1970s, with a particular focus on how the agency navigated north-south tensions in the globalization of health care and politics.   

5 As Natalie Porter (2017) has shown in her study of bird flu interventions in Vietnam, approaches based in international organisations and collaborations that seek to protect humans and animals also engage in the prioritization of “[…] the vitality of some species while at the same time marginalizing others” (p. 148).

6 Also, a series of conferences ran simultaneously, focusing specifically on different problems related to the use and study of nonhuman primates in laboratory settings from 1965 to 1967. With the WHO as organizer or co-organizer, these conferences focused specifically on different problems related to the use and study of primates in laboratory settings. Entitled the *International Symposium on Infections and Immunosuppression in Subhuman Primates*, held in London in 1965, in Lyon in 1967, and in Rijswijk in 1969, the symposia were oriented around different issues related to comparative medicine and communicable diseases of primates, new scientific insights in bacterial and viral infections, the role of environmental and experimental conditions for primate research, and hazards related to immunosuppression.

7 Evans, D.G. Dated 15 December 1969, pp. 1–4; “Hazards of Infections from Laboratory Primates—1969”, Folder V5-180-7(B), Box A.1596, M BUILD—01/01/1968-01/01/1971—The WHO Centralized Archives, Geneva, Switzerland; hereafter Folder B of Document for 22nd Expert Committee on Biological Standardization.

8 Beveridge, W. Dated 20 June 1968. Document for 22nd Expert Committee on Biological Standardization.

9 Roth, H. H. Dated 18 July 1968. Folder B of Document for 22nd Expert Committee on Biological Standardization.

10 Outschoorn, A. Dated 5 August 1968. Folder B of Document for 22nd Expert Committee on Biological Standardization.

11 Outschoorn, A. Dated 5 August 1969. Folder B of Document for 22nd Expert Committee on Biological Standardization.

12 Outschoorn, A. “Preservation of supplies of non-human primates.” Dated 9 May 1969. Folder B of Document for 22nd Expert Committee on Biological Standardization.

13 Perkins, F.T. Dated 26 July 1968, p. 1. Folder B of Document for 22nd Expert Committee on Biological Standardization.

14 Perkins, F.T. Dated 26 July 1968, p. 2. Folder B of Document for 22nd Expert Committee on Biological Standardization.

15 Perkins, F.T. Dated 26 July 1968, p. 5. Folder B of Document for 22nd Expert Committee on Biological Standardization.

16 Perkins, F.T. Dated 26 July 1968, p. 1. Folder B of Document for 22nd Expert Committee on Biological Standardization.

17 Beveridge, W. Dated 21 June 1968. Memorandum. Folder B of Document for 22nd Expert Committee on Biological Standardization.

18 Roth, H. H., 1969, p. 2; “Hazards of Infections from Laboratory Primates—1969”, Folder V5-180-7(A), Box A.1596, M BUILD—01/01/1968-01/01/1971—The WHO Centralized Archives, Geneva, Switzerland; hereafter Folder A of Document for 22nd Expert Committee on Biological Standardization.

19 Roth, H. H., 1969, pp. 1–2. Folder A of Document for 22nd Expert Committee on Biological Standardization.

20 Roth, H. H., 1969, p. 2. Folder A of Document for 22nd Expert Committee on Biological Standardization.

21 Roth, H. H., 1969, p. 8. Folder A of Document for 22nd Expert Committee on Biological Standardization.

22 Cohen, H. Dated 8 April 1969. Folder B of Document for 22nd Expert Committee on Biological Standardization.

23 Evans, D.G. Dated 15 December 1969, p. 2. Consultant Report for WHO. Folder B of Document for 22nd Expert Committee on Biological Standardization. Examples of institutions and corporate actors that sent in comments are Paul-Ehrlich Institute, Frankfurt Germany, Yerkes Regional Primate Center, USA; National Institute of Health (NIH), Bethesda, Maryland, USA; Delta Regional Primate Research Center, USA; Cornell Medical University, USA; Food and Agricultural Organization, International Council for Laboratory Animals (ICLAS), Allington Farms, UK; Medical Research Council, UK; Santoz Ltd, Switzerland; Merck, Sharpe and Dohme Research Laboratories, USA; National Biological Standards Laboratory, Australia; Radiobiological Institute of the Organization of Health Research TNO, Rijswick Netherlands; Statens Seruminstitutt, Denmark; National Institute of Public Health, Norway; National Institute of Public Health, France; The Food and Drug Directorate, Department of National Health and Welfare, Canada; Public Health Laboratories, Cairo, Egypt; Ministry of Agriculture, Nairobi; University of California, Davis, USA; The Hebrew University Medical School, Department of Medical Ecology, Israel; Royal College of Surgeons of England; Institute of Poliomyelitis and Viral Encephalitides, Moscow, USSR; Statni Veterinarni Sprava, Czech Republic; The Max Planck Institute, Germany; Jordan Vaccine Institute, Ministry of Health; The Zoological Society of London; Department of Livestock and Agriculture, Thailand. There were no voices representing African countries or countries in South America, except for the Public Health Laboratories in Cairo and the Ministry of Agriculture in Nairobi. Indeed, a representative from Uganda would be part of the scientific committee that finalized the Technical Report, and on the list of those that were invited to submit comments was the Institute of Veterinary Medicine at the University of Dakar in Senegal.

24 Again see Kirk’s (2019) study of the Silver Spring monkey controversy in the early 1980s. In his paper he shows how the Silver Spring controversy was not merely an event of society-science polarization—but rather an event where two distinct cultures of care met.

25 Trum, B. F. Undated [1969]. Folder B of Document for 22nd Expert Committee on Biological Standardization. In folder B, several newspaper and journal articles were archived together with the expert documents and correspondence that demonstrated the brute headlines that now were increasingly emerging in the public. For instance, “Scientists peril monkey populations” (Atlanta Journal-Constitution, July 3, 1968. http://philsci-archive.pitt.edu/archive/00003390. Accessed 26 June 2007) and “The Overkill” (Roth 1968).

26 Trum, B. F. Undated [1969]. Folder B of Document for 22nd Expert Committee on Biological Standardization.

27 Arthur Riopelle was an experimental psychologist, and before he started at the regional primate center, he had worked at the Psychology Division of the U.S. Army Medical Laboratories at Fort Knox, and the Yerkes Laboratories of Primate Biology, in Florida. He had also been involved in a project that involved launching monkeys into space (Mason 2013).

28 Riopelle, A. Undated [1969]. Folder B of Document for 22nd Expert Committee on Biological Standardization.

29 Ibid.

30 Riopelle, A. Undated [1970]. Folder B of Document for 22nd Expert Committee on Biological Standardization.

31 Cohen, H. Dated 8 April 1969. Folder B of Document for 22nd Expert Committee on Biological Standardization.

32 Ibid.

33 Roth, H.H. Dated 18 July 1968. Folder B of Document for 22nd Expert Committee on Biological Standardization.

34 See Angela Cassidy’s (2018) piece for more about the contradictory aspects of what is now known as One Health. In her piece, she argues for how animal health issues related to disease transmission and as experimental models for human disease “rebrands longlasting research agendas that are more concerned with the health of humans than that of animals” (abstract). See also Mette Nordahl Svendsen (2017) on Pigs in Public Health about what it means to consider pigs in relation to human health in this context.

35 While there were ideal standards available, local differences were inescapable because of economic and technical circumstances across transnational networks of laboratories and producers (see Druglitrø 2014; see also Druglitrø 2016 for an account on the development of local standards for housing and caring for rhesus macaques at the Public Health Institute in Norway).

36 Roth, H.H. Dated 18 July 1968. Folder B of Document for 22nd Expert Committee on Biological Standardization

37 Ibid.

38 Bowen, W. H. Dated 21 April 1969. Folder B of Document for 22nd Expert Committee on Biological Standardization.

39 Schmidt, L. H. Dated 31 March 1969. Folder B of Document for 22nd Expert Committee on Biological Standardization.

40 Bourne, G. Dated 2 June 1970. Folder B of Document for 22nd Expert Committee on Biological Standardization.

41 The Hebrew University Medical School, Department of Medical Ecology, Jerusalem. Dated 6 March 1969. Folder B of Document for 22nd Expert Committee on Biological Standardization.

42 Balner, H. 1969. Dated 8 April 1969. Folder B of Document for 22nd Expert Committee on Biological Standardization.

43 P.B. Stones, Pfizer group. Dated 11 March 1969. Folder B of Document for 22nd Expert Committee on Biological Standardization.

44 Shamrock Farms. Dated 18 September 1969. Folder B of Document for 22nd Expert Committee on Biological Standardization.

45 P.B. Stones, Pfizer group. Dated 11 March 1969. Folder B of Document for 22nd Expert Committee on Biological Standardization.

46 Shamrock Farms. Dated 18 September 1969. Folder B of Document for 22nd Expert Committee on Biological Standardization.

47 Ibid.

48 Ibid.

49 Balner, H. Dated 8 April 1969. Folder B of Document for 22nd Expert Committee on Biological Standardization.

50 Thomas, A.K. Dated April 1969. Folder B of Document for 22nd Expert Committee on Biological Standardization.

51 WHO. Dated 27 January 1970. Memorandum: Non-human primates for biomedical purposes. Folder A of Document for 22nd Expert Committee on Biological Standardization.

52 Dr. Ploog. Dated 15 April 1969. Document for 22nd Expert Committee on Biological Standardization.

53 Evans, D.G. Dated 15 December 1969, p. 3. Folder B of Document for 22nd Expert Committee on Biological Standardization.

54 Schmidt, L. H. Dated 31 March 1969. Folder B of Document for 22nd Expert Committee on Biological Standardization.

55 Ibid.

56 See Kirk (2019); for more on the “culture of care” in animal research, see Davies et al. (2018).

57 See also Svendsen (2017), Woods et al. (2018).
